# Supplementary material for: Morphology-Dependent Interactions between α-Synuclein Monomers and Fibrils
Source: Int J Mol Sci. 2023 Mar 8;24(6):5191. doi: 10.3390/ijms24065191 (PMC10049171; doi:10.3390/ijms24065191)
Supplement: Supplementary file 1 [file ijms-24-05191-s001.zip › ijms-2191221-supplementary.pdf]

Supporting Information

Morphology-Dependent Interactions between α-Synuclein Monomers and Fibrils

Tinna Pálmadóttir[1], Chris Waudby[2], Katja Bernfur[1], John Christodoulou[2], Sara Linse[1], Anders Malmendal[1,3]

[1] Biochemistry and Structural Biology, Lund University, P. O. Box 124, SE-221 00, Lund, Sweden.

[2] Institute of Structural and Molecular Biology, University College and Birkbeck College, London WC1E 7HX, United Kingdom

[3] Department of Science and Environment, Chemistry, Roskilde University, P.O. Box 260 DK-4000 Roskilde, Denmark.

*Corresponding authors.

Tinna Pálmadóttir, e-mail: tinna.palmadottir@biochemistry.lu.se

Anders Malmendal, e-mail: amalm@ruc.dk

Sara Linse, e-mail: sara.linse@biochemistry.lu.se

1. Verification of the expression of α-synuclein

The expression of α-synuclein was performed as explained in section 2.1. Prior to harvesting, 1 mL samples were taken from the cultures and centrifuged at 20000 g at room temperature. The supernatant was discarded, and the pellets were frozen at -20°C. Frozen pellets were taken out from the freezer and each dissolved in 100 µL sterile H_2O. Thereafter, the samples were boiled for 2 minutes and centrifuged again for 10 min at 20000 g. Supernatants were analyzed with SDS-PAGE using Novex™ 10-20% Tricine gels (Invitrogen by Thermo Fisher Scientific) (Figure S1).

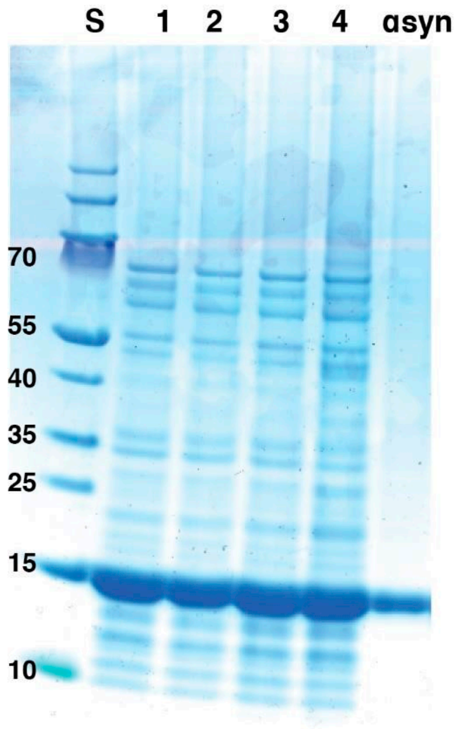

Figure S1: Testing of expression of α-synuclein in E. coli. *Expression test samples of 1 mL were taken from four different baffled flask containing cultures originating from four individual colonies. Welsl 1-4, show the supernatant after boiling and the second centrifugation. The strong band appearing around 15 kDa contains α-synuclein. Well 5 shows a pure alphasynuclein as a reference.*

2. Evaluation of the mass and the purify after purification of α-synuclein using Mass Spectrometry.

After purification of α-synuclein (explained in section 2.1), the samples were analyzed with MALDI Mass Spectrometry to evaluate the purify of the samples as well as to confirm that the protein has the correct mass (unmodified). A monomeric sample was also analyzed after the size exclusion chromatography step explained in section 2.2. Figure S2 shows pure protein of correct mass.

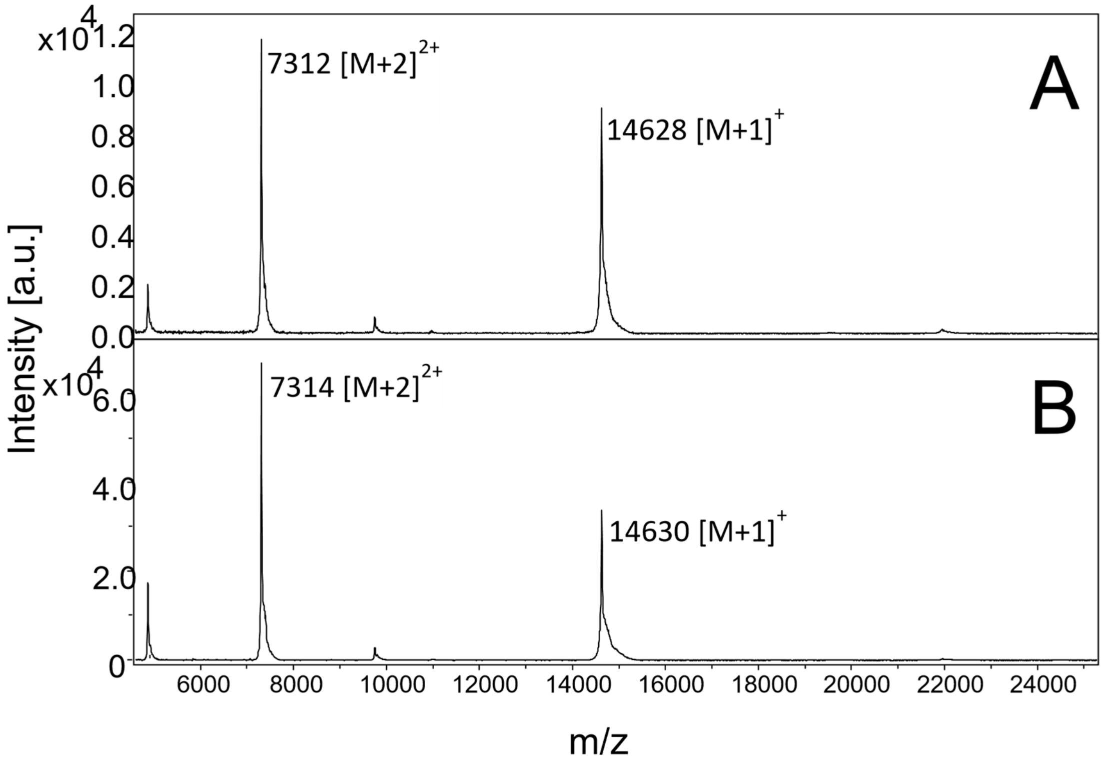

Figure S2. Intact MALDI mass spectra in linear mode of purified ^{15}N α-synuclein. *A. before size exclusion chromatography and **B**. the monomeric fraction after size exclusion chromatography. Peaks arising form singly charged ($[M+1]^{+}$) and doubly charged ($[M+2]^{2+}$) α-synuclein monomers are labelled in the spectra.*

3. Comparison of CD spectra obtained for different α-synuclein fibril samples

CD-spectra were obtained for 17 independent α-synuclein fibril samples formed at the same condition. The spectra can be divided into two groups, one with peaks at lower wavelengths (morphology A) and the other one with the peaks shifted to higher wavelengths (morphology B)

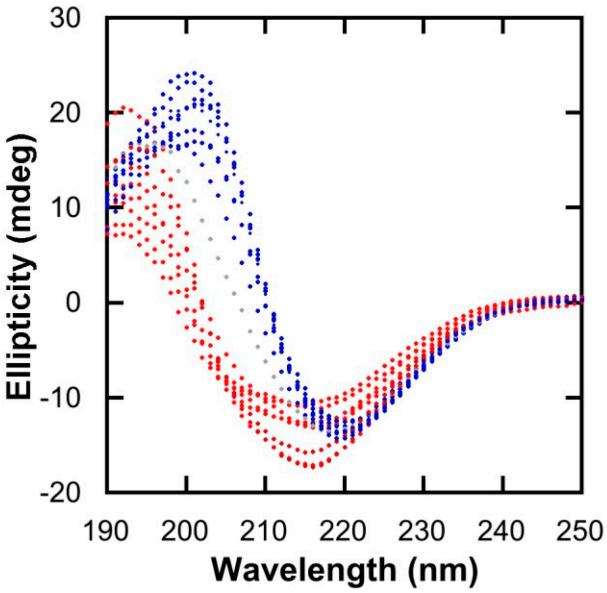

Figure S3: CD spectra obtained for 17 independent α-synuclein fibril samples formed at the same conditions. *All samples contain 70 uM α-synuclein at pH 7. The CD spectra can be grouped into two groups depending on the wavelength of the peaks, red (morphology A) and blue (morphology B). There is one sample that cannot be grouped into one of the groups (gray).*

4. The effect of solubility on the CD spectra of morphology A and B

As mentioned in section 3.1, we tested whether the difference in monomeric concentration present in fibril samples of morphology A and B could be the reason for the difference in the CD spectra between the two morphologies (the shift of the peaks). This was done by calculating the theoretical spectra for the samples containing only fibrils (without monomers in equilibrium with the fibrils) using experimental spectra obtained for the monomeric sample and the fibrillar samples of morphology A and B (Figure S4 a and b), and the average concentration of monomers present in samples of morphology A and B, 24.4 and 6.5 μM, respectively, as measured by NMR (section 3.1 and 3.2). In addition, CD spectra were taken of a fibril sample (morphology A) mixed with a monomeric sample at different ratios (Figure S4c). The results showed that the shift in the β-sheet signal between the two morphologies cannot be explained by the difference in solubility between the two morphologies.

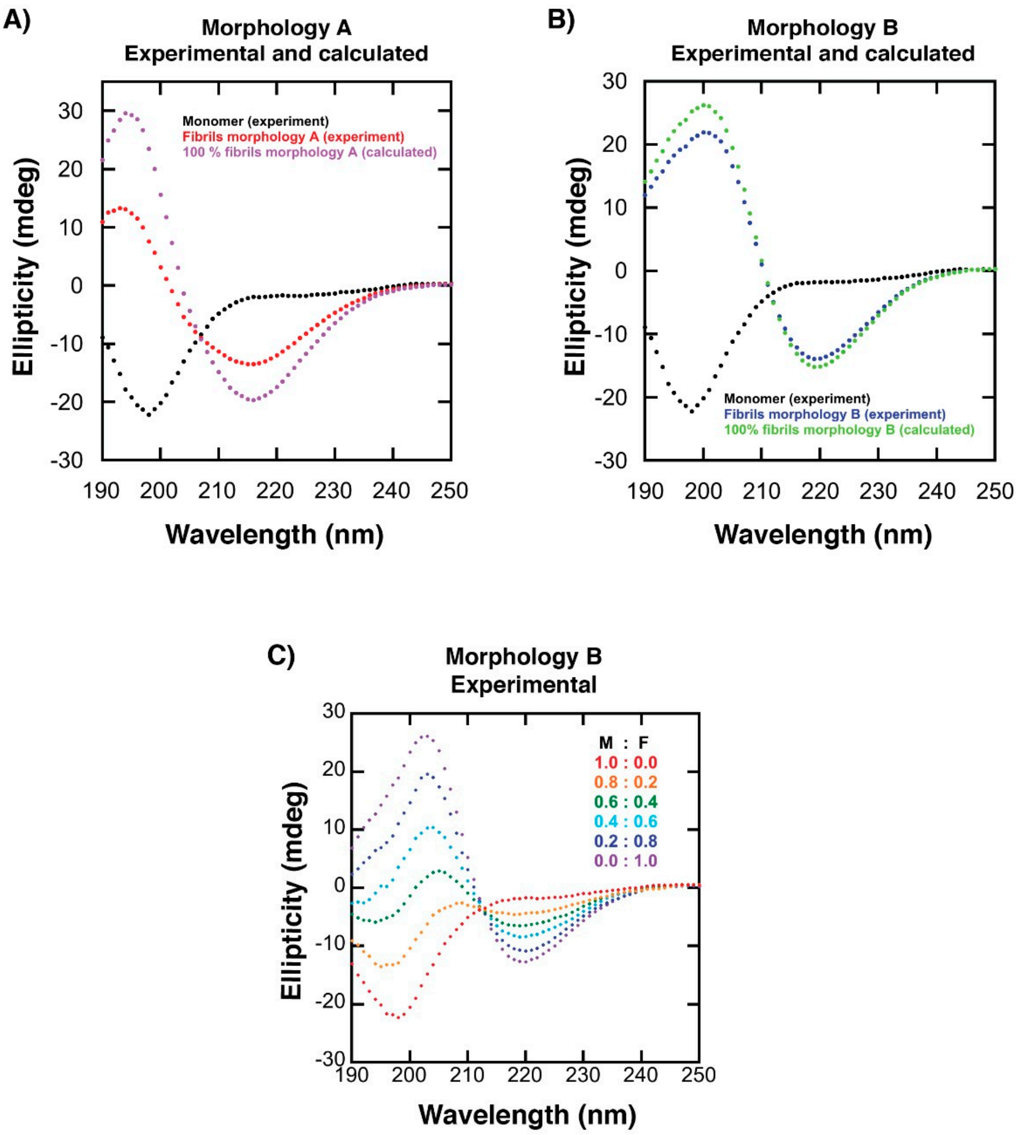

Figure S4: Different concentration of soluble monomer in presence of fibrils does not cause a shift in the position of the beta-sheet peaks. (A). *Experimental CD spectra of monomeric sample and fibril sample (morphology A) and a calculated CD spectrum of a sample only containing fibrils.* ***(B).*** *Experimental CD spectra of monomeric sample and fibril sample (morphology B) and a calculated CD spectrum of a sample only containing fibrils.* ***(C)*** *Effect of different ratios of monomeric and fibril samples on the CD spectra. Monomers and Fibrils were prepared as explained in methods. A sample containing fibrils (morphology B) in equilibrium with monomers were mixed with monomeric sample in different ratios, monomeric sample (M) : Fibril sample (F); 1.0:0.0; 0.8:0.2; 0.6:0.4; 0.4:0.6; 0.2:0.8; 0.0:1.0. CD spectra were taken at each ratio and compared.*

5. Chemical shift changes induced in the presence of fibrils

While the fibril induced ^{15}N chemical shift changes of the monomer in the presence of morphology A are limited, those of in the presence of morphology B are larger. Many of the residues from A124 and onwards show large chemical shift perturbations.

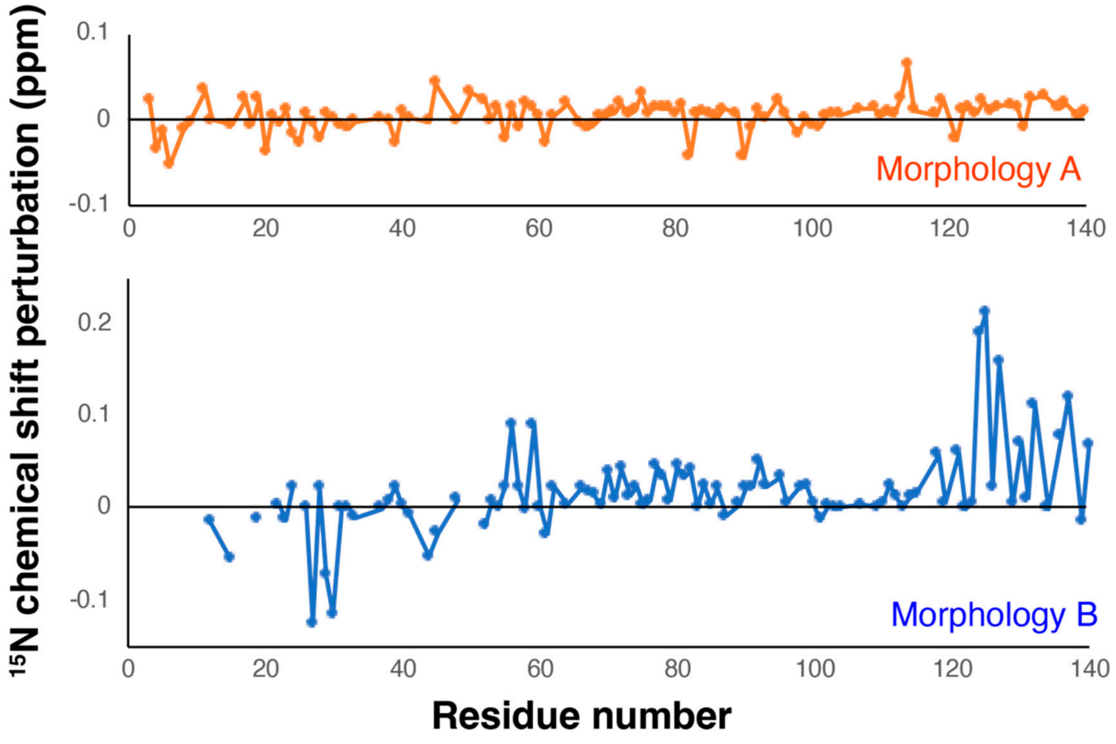

Figure S5:** ^{15}N **chemical shift changes induced in the presence of fibrils of morphology A (red) and B (blue).

6. Evolution of morphology A and B with increased incubation time

Figure S6 shows the same data as is shown in Figure 4 (section 3.3.2.), but represented in a different way. Here, the CD spectra taken of morphology A and B at each time point are compared in the same graph. The different graphs show the evolution of the CD spectra after 1, 2, 3, and 6 days of incubation. It shows clearly how the CD spectra of fibrils of morphology A and B differ after 1 day incubation and after 6 days of incubation they have become nearly identical.

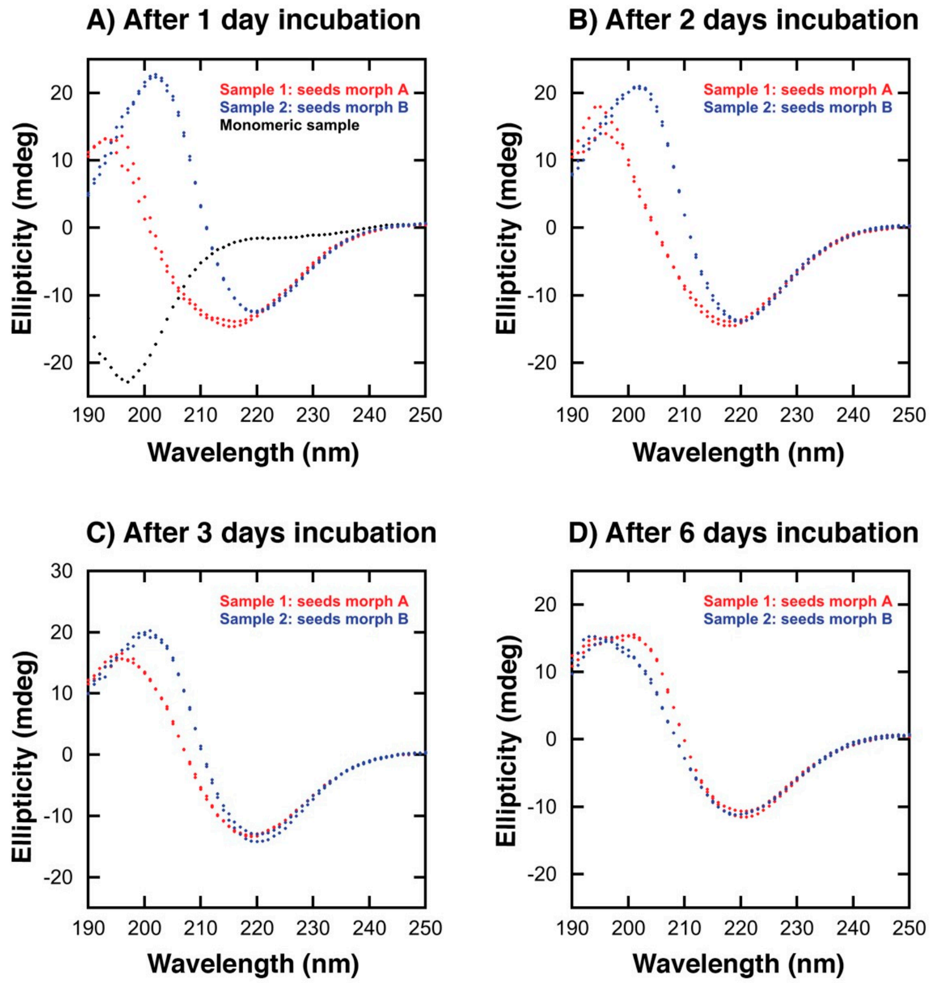

Figure S6: The replication of morphologies A and B using 1% seed. *The different graphs show CD spectra of monomeric sample (black), supplemented 1% seeds of morphology A (red), 1% seeds of morphology B (blue) (A) Samples measured after 1 day of incubation. The CD spectrum of monomeric sample before incubation at 37°C (time point zero) is shown in black. (B) Samples measured after 2 days of incubation. (C) Samples measured after 3 days of incubation. (D) Samples measured after 6 days of incubation.*

7. Monomer concentration over time as measured by MALDI-MS.

Over time samples with fibrils of morphology A or B seem to equilibrate to the same free monomer concentration. The quantification by MALDI-MS shown here are in full agreement with those observed by SDS-PAGE, see Figure 5.

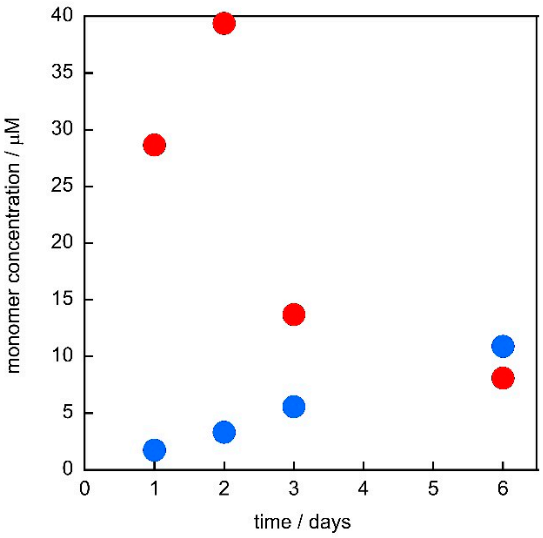

Figure S7: Monomer concentration over time. *The monomer concentration in samples that initially appeared as morphology A (red dots) or B (blue dots) was measured using MALDI MS after isotope spiking and tryptic digestion for the same samples as analyzed by SDS PAGE (see Figure 5).*

8. Binding of Thioflavin T to fibrils of morphology A and B

As presented in section 3.4 and explained in section 2.5, surface properties of morphologies A and B were investigated by measuring the ThT fluorescence emission spectra of the samples, titrated with ThT. Here we show the full emission spectra of each sample (morphology A and B, in duplicate) recorded during titration with ThT.

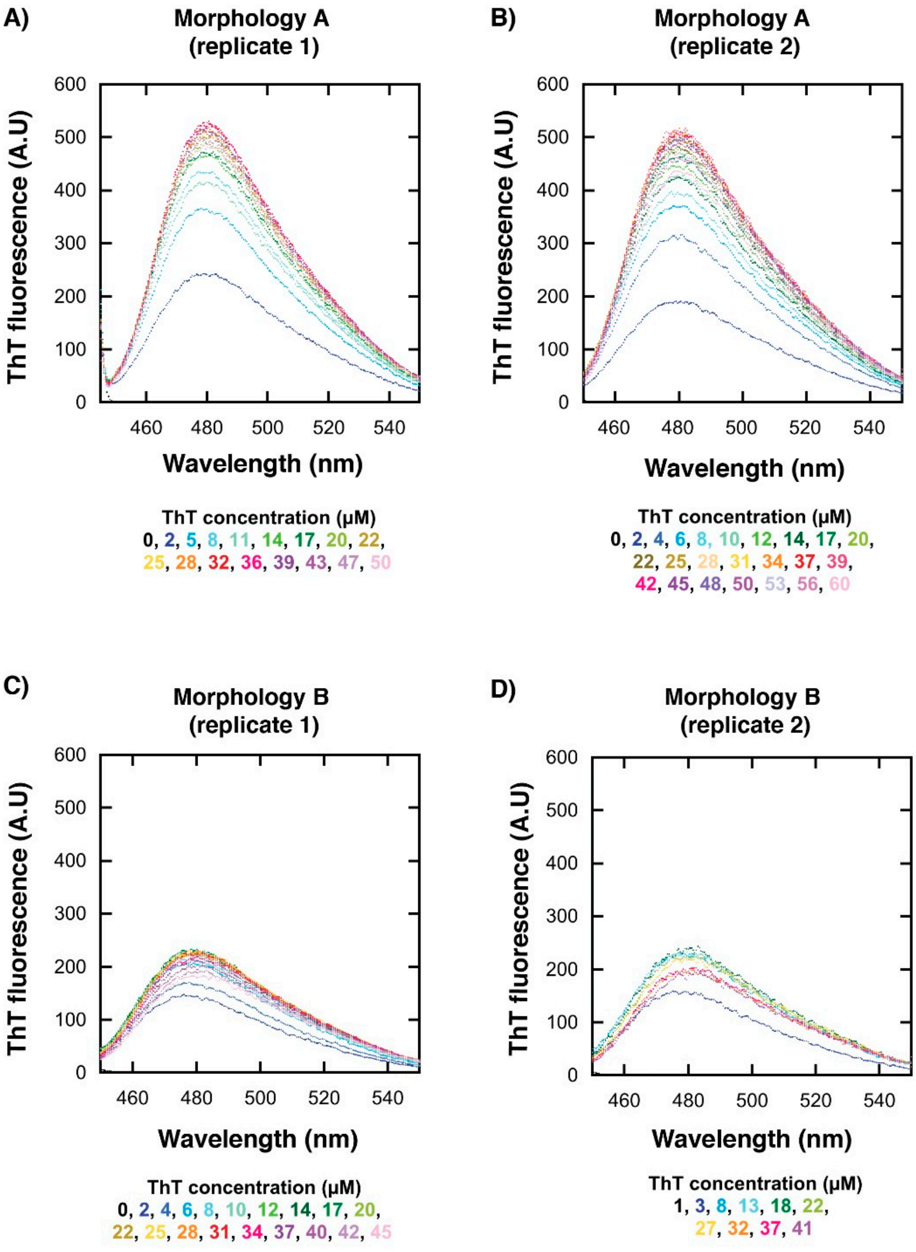

Figure S8: ThT fluorescence emission spectra of morphology A and B. *ThT was titrated into sample containing fibrils of morphology A or B. Two independent samples of each morphology were investigated. The samples were excited at 440 and the emission spectra were recorded between 450 to 550 nm. The final concentration of ThT after titration into the samples is shown in different colors (see below each graph).*
